# Intimate Partner Violence after Disclosure of HIV Test Results among Pregnant Women in Harare, Zimbabwe

**DOI:** 10.1371/journal.pone.0109447

**Published:** 2014-10-28

**Authors:** Simukai Shamu, Christina Zarowsky, Tamara Shefer, Marleen Temmerman, Naeemah Abrahams

**Affiliations:** 1 Gender and Health Research Unit, Medical Research Council, Cape Town, South Africa; 2 International Centre for Reproductive Health, Ghent University, Ghent, Belgium; 3 School of Public Health, University of the Western Cape, Cape Town, South Africa; 4 Women's and Gender Studies, University of the Western Cape, Cape Town, South Africa; 5 University of Montreal Hospital Research Centre (CR-CHUM), Montreal, Canada; University of Washington, United States of America

## Abstract

**Background:**

HIV status disclosure is a central strategy in HIV prevention and treatment but in high prevalence settings women test disproportionately and most often during pregnancy. This study reports intimate partner violence (IPV) following disclosure of HIV test results by pregnant women.

**Methods:**

In this cross sectional study we interviewed 1951 postnatal women who tested positive and negative for HIV about IPV experiences following HIV test disclosure, using an adapted WHO questionnaire. Multivariate regression models assessed factors associated with IPV after disclosure and controlled for factors such as previous IPV and other known behavioural factors associated with IPV.

**Results:**

Over 93% (1817) disclosed the HIV results to their partners (96.5% HIV− vs. 89.3% HIV+, p<0.0001). Overall HIV prevalence was 15.3%, (95%CI:13.7–16.9), 35.2% among non-disclosers and 14.3% among disclosers. Overall 32.8% reported IPV (40.5% HIV+; 31.5% HIV− women, p = 0.004). HIV status was associated with IPV (partially adjusted 1.43: (95%CI:1.00–2.05 as well as reporting negative reactions by male partners immediately after disclosure (adjusted OR 5.83, 95%CI:4.31–7.80). Factors associated with IPV were gender inequity, past IPV, risky sexual behaviours and living with relatives. IPV after HIV disclosure in pregnancy is high but lower than and is strongly related with IPV before pregnancy (adjusted OR 6.18, 95%CI: 3.84–9.93).

**Conclusion:**

The study demonstrates the interconnectedness of IPV, HIV status and its disclosure with IPV which was a common experience post disclosure of both an HIV positive and HIV negative result. Health services must give attention to the gendered nature and consequences of HIV disclosure such as enskilling women on how to determine and respond to the risks associated with disclosure. Efforts to involve men in antenatal care must also be strengthened.

## Introduction

HIV is an infectious disease and status disclosure to affected and potentially infected sexual partners is a central strategy in HIV prevention and treatment [1]. Globally, encouraging HIV status disclosure dates back to the late 1980s, modelled on the public health practice of partner notification [2]. The benefits of disclosure are well documented and include helping to motivate partners to seek HIV testing, reducing risky sexual behaviour and making informed and healthy choices to reduce HIV transmission [3]. However, disclosure is a complex and a gendered phenomenon. In high prevalence settings women test disproportionately [4], often during pregnancy, and are expected to disclose to sexual partners. Given the relationship between intimate partner violence (IPV) and gender inequality [5], [6], disclosure may have unintended consequences such as the extension of IPV during pregnancy, particularly in relationships with previous abuse. Possible negative consequences after HIV disclosure were reported in Africa in the early 1990's when antiretroviral drugs were not available in Africa [7]. Disclosure is highly emotionally charged; more than simply conveying medical information to a partner, it raises questions of trust, loyalty and faithfulness [8]. Disclosure is therefore much more difficult for women in relationships where decisions are male dominated.

Research on HIV disclosure has been uneven- both in time and geography. For example, several studies have been published in the early 2000's with little further research until very recently. African studies on outcomes of disclosure present different findings, with some reporting positive outcomes including being accepted, receiving social support including treatment access and adherence and increased opportunities for risk reduction, [6], [9], while others report negative outcomes such as stigma and discrimination [10], [11]. Studies that assessed negative outcomes did not specifically focus on IPV. Gender inequality, social demographics and HIV are important risk factors for IPV [12]–[14]. However, our understanding of these factors in relation to IPV after disclosure is still limited.

A global review conducted by Maman and colleagues showed that although 26 out of 31 studies reported negative outcomes after disclosure, violence was not commonly reported but also not precisely measured [3]. The only study conducted in Zimbabwe of outcomes of disclosure, on a non-random sample of postnatal women in an urban setting (n = 221), reported 8% of women experiencing physical violence after disclosure [15]. Two reviews on disclosure rates and outcomes concluded that it was difficult to assess the extent of negative outcomes as there were often no data on the previous state of relationships [8], [11]. This is particularly relevant for IPV since it is important to understand HIV disclosure and IPV, whether IPV after disclosure is an extension of previous violence or is specifically associated with the HIV test. A review of African studies revealed a decrease in violence during pregnancy by 10% [16]. However, this review did not assess the dynamics of violence after disclosure. This study assessed whether a decrease or increase of violence happens after HIV disclosure. Studies often did not separate outcomes by HIV status and the few that did showed contrasting results [3], [9]. HIV testing has become an integral part of ante-natal care in high HIV prevalence settings such as Zimbabwe. This paper presents prevalence of HIV disclosure - positive or negative results - to an intimate partner during pregnancy as well as factors associated with IPV after disclosure of HIV test results.

### Study Context

Women of childbearing age in Zimbabwe experience high prevalence of both violence and HIV. The Zimbabwe Demographic and Health Survey [17] found 40% women and a third (33%) of men justifying partner beating. It also found 43.4% women reporting being ever physically or sexually abused, with intimate partners perpetrating 75% of the physical violence and 82% of sexual violence. Only 4% women in Zimbabwe test for HIV before pregnancy while 65% of the pregnant women test for HIV through the voluntary counselling and testing and 99.9% test through the provider-initiated HIV testing approach which is current government policy [15]. Although women are encouraged to test with their partners during antenatal care, men rarely accompany their partners for HIV tests and antenatal care [18].

## Methods

A cross sectional survey was conducted among women attending a 10-day or six-weeks postpartum clinic in six public clinics in low-income urban areas of Harare, Zimbabwe between May and September 2011. Women aged 15 to 49 queuing for postnatal care were invited for face-to-face interviews asking closed questions in the local language (Shona) by trained female fieldworkers in a private space. With their permission, participants' HIV test results were obtained from antenatal clinic records. We did not get information on whether women tested with their partners although they were encouraged to do so by health workers. We approached 2101 women and interviewed 2042 (97% response rate). The overwhelming majority of women had tested for HIV and results were available for 95.5% (N = 1951). Detailed methodology and overall findings including on IPV during pregnancy and HIV risk have been reported elsewhere [16].

Participants were asked whether and how soon they disclosed their test results to their partners, and about their partners' immediate reactions after disclosure. This latter question had a wide range of response options including positive responses such as “he was happy” or “supportive” and negative responses such as threat to end relationship, blaming woman's past sexual life, labelling her a prostitute, experiences of and threats of violence. Our qualitative research informed the development of the question and response options. We used an adapted WHO questionnaire on gender-based violence to measure violence in this study [19]. Physical, sexual and emotional IPV were measured using six, three and four questions respectively and we further specifically asked if these experiences followed disclosure of the HIV test result during the most recent pregnancy. The violence measure referred to the period after disclosure up to the end of the pregnancy. We measured IPV up to the end of the pregnancy so that we could achieve an equal comparison among our participants who had delivered their babies within 10 days to 6 weeks after delivery.

The study included variables found in research to be associated with IPV and HIV as well as potential confounders. Past IPV was measured by asking respondents about experiences of IPV in the 12 months before the pregnancy. Respondents were further asked about their experiences of physical and sexual abuse before age 15, risky sexual behaviour such as woman ever engaging in transactional sex, and partner's history of sexually transmitted infections (STI) (whether partner tested positive for STI before). They were also asked about the number of pregnancies they ever had and whether they ever tested for HIV before the most recent HIV test in antenatal care. Male partner violent behaviours were assessed by asking the respondent if her partner ever fought with another man since she partnered with him. Partner controlling behaviour (Cronbach alpha 0.60) and sexual abuse attitudes (Cronbach alpha 0.69) were measured using six behaviours and attitudes respectively, as used in previous research [20]. Binary variables were created with zero to two behaviours/attitudes described as none/low partner control/sex abuse attitude and 3–6 behaviours/attitudes representing high-level partner control/sexual abuse attitudes.

### Data Analysis

Data were analysed using Stata version 12 (StataCorp 2009). Prevalence of HIV and IPV forms (physical, sexual, emotional and combined forms) were calculated. We also calculated the proportion of women who reported no previous abuse but reported abuse for the first time after disclosure. We assessed IPV and HIV status and constructed an ordered IPV variable, with never experienced physical, sexual or emotional abuse, a single type of IPV, two types and three or more types of violence and used this as the outcome in the multivariate analysis of factors associated with IPV after disclosure of HIV test results. An ordered variable with four categories allowed assessing violence frequency. After assessing candidate variables at the univariate level, a generalised ordered multiple, stepwise regression analysis was done adjusting for woman's age, education, marital status, past violence, whether woman tested for HIV before, total number of previous pregnancies, time of testing for HIV and time of interview, the latter because some women tested in the first trimester while others in the last trimester thus affecting the duration of exposure and measurement of disclosure and violence after disclosure. The regression model compared the effect of medium (2 types) to higher (3 or more types) with no or lower (0–1 type) IPV. The generalized ordered logit/partial proportional odds model for ordinal dependent variables [21] was fitted with all variables using gologit2 command and we used the backward elimination approach to remove insignificant variables at the 10% level. The final model was the best fit model with the lowest log likelihood ratio. We tested the proportional odds or parallel-lines assumption using a Wald test which was insignificant (p = 0.6872) showing no violation of the proportional odds/parallel lines assumption.

An additional logistic regression model was tested for the association between partner's reaction after disclosure (0 = positive response, 1 = negative response) and women's HIV status, controlling for past violence and demographic factors (age, education and marital status).

Ethics approval was obtained from the Medical Research Council of Zimbabwe and the University of the Western Cape. Written informed consent was sought and provided before interviewing participants. For women under 18 years of age, the next of kin, caretaker or guardian was asked to provide written consent before the client provided written assent to participate in the study. Women were provided with information about organisations that they could consult for counselling and support if needed. The study followed the WHO ethical guidelines for researching violence against women and girls [22].

## Results

**Figure 1:**
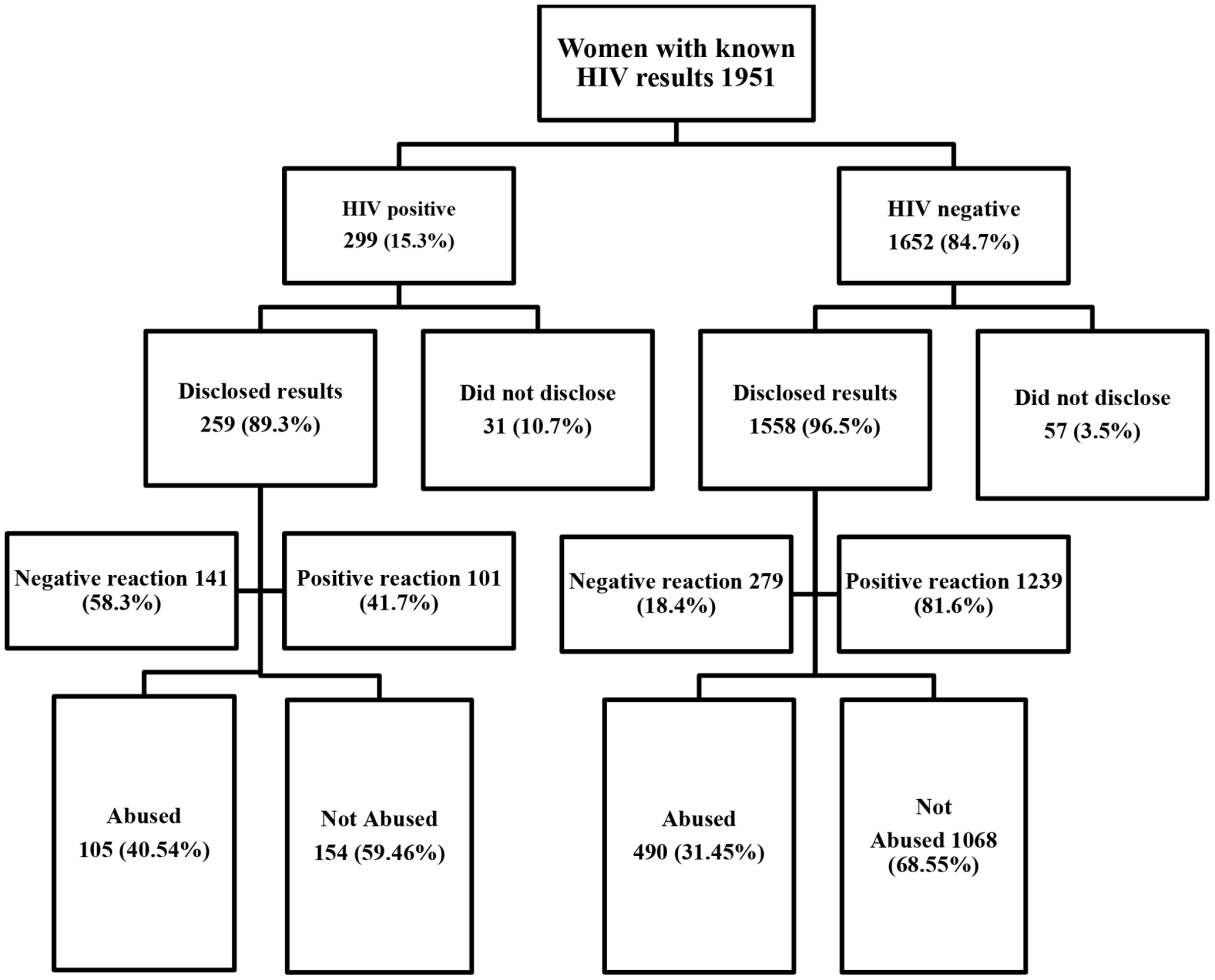
Prevalence of intimate partner violence (physical, sexual or emotional) after testing and disclosing HIV status to a partner (%).

Among the 1951 women included in the study, the majority disclosed their HIV test results (93.1%, N = 1817) to their partners (Figure 1). 97.2% of the women reported disclosing their results within three days of receiving their results. Overall HIV prevalence was 15.3% (95% CI: 13.7 16.9) (Figure 1). HIV prevalence among women who did not disclose (35.2%, 95% CI: 25.0–45.4) was more than double that among women who disclosed to their partners (14.3%, 95% CI: 12.6–15.8). One in ten of the HIV positive women (10.7%) did not disclose compared to 3.5% of the HIV negative women (p<0.0001). Nearly one in four women (23.9%) reported a negative reaction by their partner immediately after disclosure, such as threats of or actual violence. More women experienced a negative reaction if they tested positive than if they tested negative (58.3% vs. 18.4% p<0.0001). See Figure 1. HIV positive women were nearly six times more likely to report a negative reaction from the partner compared to HIV negative women (OR: 5.83 95% CI: 4.31–7.90).

Overall, nearly a third (32.8%) of women who disclosed reported some form of abuse that took place any time between disclosure and delivery with higher rates among HIV positive women (40.5%) than HIV negative women (31.5%) (p = 0.004) (Unadjusted OR: 1.48 95% CI: 1.13–1.94).

Over 60% of women reported at least one episode of physical, sexual or emotional IPV in the 12 months before pregnancy as presented in Table 1. IPV in the 12 months before the pregnancy was higher than IPV after disclosure for all types of violence. Higher levels of sexual (22.6%) and emotional IPV (18%) than physical IPV (5.8%) were reported after disclosure. As noted above, HIV positive women were much more likely to report a negative reaction to disclosure. In addition, significant differences between women reporting and those not reporting IPV after disclosure by HIV status were found for most types of IPV after disclosure (See Table 1).

**Table 1:** Prevalence of intimate partner violence 12 months before pregnancy and after HIV disclosure by HIVsero-status.

Violence type	HIV negative	HIV positive	All women	p-value
	n = 1558	n = 259	n = 1817	
	(%)	(%)	(%)	
**IPV BEFORE PREGNANCY**				
**Physical: No**	1239 (79.5)	199 (76.8)	1438 (79.1)	
**Yes**	319 (20.5)	60 (23.2)	379 (20.9)	0.324
**Sexual: No**	1030 (66.1)	157 (60.6)	1187 (65.3)	
**Yes**	528(33.9)	102 (39.4)	630 (34.7)	0.085
**Emotional: No**	954 (61.2)	141 (54.4)	1095 (60.3)	
**Yes**	604 (38.8)	118 (45.6)	722 (39.7)	0.039
**Physical or sexual: No**	866 (55.6)	132 (51.0)	998 (54.9)	
**Yes**	692 (44.4)	127 (49.0)	819 (45.1)	0.167
**Physical, sexual or emotional: No**	619 (39.7)	97 (37.5)	716 (39.4)	
**Yes**	939 (60.3)	162 (62.6)	1101 (60.6)	0.487
**IPV AFTER DISCLOSURE**				
**Physical: No**	1478 (94.9)	233 (90)	1711(94.27)	
**yes**	80 (5.1)	26 (10.0)	106 (5.8)	0.002
**Sexual: No**	1214 (77.9)	192 (74.1)	1406 (77.4)	
**Yes**	1344 (22.1)	67 (25.9)	411 (22.6)	0.177
**Emotional: No**	1301 (83.3)	187 (72.2)	1488 (81.9)	
**Yes**	1257 (16.5)	72 (27.8)	329 (18.1)	<0.0001
**Physical or sexual: No**	1175 (75.4)	183 (70.7)	1358 (74.7)	
**Yes**	1383 (24.6)	76 (29.3)	459 (25.3)	0.103
**Physical or emotional: No**	1280 (82.2)	183 (70.7)	1463 (80.5)	
**Yes**	278 (17.8)	76 (29.3)	354 (19.5)	<0.0001
**Sexual or emotional violence: No**	1080 (69.3)	155 (59.9)	1235(68.0)	
**Yes**	478(30.7)	104 (40.2)	582 (32.0)	0.002
**Physical, sexual or emotional: No**	1068 (68.6)	154 (59.5)	1222 (67.3)	
**Yes**	490 (31.5)	105 (40.5)	595 (32.8)	0.004

N = 1817.

A total of 595 (32.8%) experienced at least one form of abuse after disclosure (N = 1817). Of these, 68 (11.4%) women reported experiencing IPV for the first time only after disclosure and a significant proportion of them (22.1%) had tested HIV positive.

Table 2 shows the socio-demographic characteristics of the sample against a number of IPV acts after disclosing HIV test results. Significant differences were found for the following variables: age, reporting partner controlling behaviour, child abuse, engaging in transactional sex, testing positive to STI or HIV, experiencing IPV in the 12 months before pregnancy, having a partner who used violence on another man, and if a woman was ever injured by a partner.

**Table 2:** Characteristics of participants by experiences of physical, sexual and/or emotional intimate partner violence after HIV disclosure.

	IPV experiences				
Variables	No IPV (%)	1 IPV event (%)	2 IPV events (%)	3+ IPV events (%)	p-value
Couple lives with woman's family member/s (vs. no) - 729/1783	543(74.5)	106 (14.5)	45 (6.2)	35 (4.8)	<0.0001
Couple lives with partner's family member/s (vs. no) (1176/1789)	863(73.4)	167 (14.2)	78(6.6)	68 (5.8)	<0.0001
Age -under 25 years vs 25+ (805/1813)	556 (69.07)	130 (16.2)	54 (6.7)	65(8.07)	0.014
Married women vs unmarried (1653/1816)	1,112 (67.3)	308(18.6)	129 (7.8)	104(6.3)	0.064
Only primary education (129/1813)	76(58.9)	79(22.5)	14 (10.9)	10(7.8)	0.201
Woman tested for HIV before vs no (905/1810)	617(68.2)	163(18.0)	74(8.2)	51(5.6)	0.290
First pregnancy vs 2+ (636/1817)	434(68.2%)	116 (18.2)	43(6.8)	43(6.8)	0.642
Experiencing 3–6 (vs. 0–2) controlling behaviours (337/1744)	185(54.9)	54(16)	37(11.0)	61(18.1)	<0.0001
Woman endorsing 3–6 (vs.0–2) sexual abuse attitudes (558/1666)	337(60.4)	130 (23.3)	55(9.9)	36(6.5)	0.002
Partner ever fought with another man (vs. no fighting) (285/1707)	171(60)	31(10.9)	38(13.3)	45(15.8)	<0.0001
Woman ever injured by a partner (vs.no) (113/1808)	49(43.4)	16(14.2)	10(8.9)	38(33.6)	<0.0001
Child physical and/or sexual abuse (vs. none) (361/1809	181(50.1)	90(24.9)	49(13.6)	41(11.4)	<0.0001
Woman stopped/discouraged from accessing antenatal care (vs. encouraged) (189/1802)	97 (51.3)	32(16.9)	26(13.8)	34(18.0)	<0.0001
Woman ever had transactional sex (vs. no) (267/1816)	134(50.2)	51(19.1)	40(15)	42(15.7)	<0.0001
Partner ever tested STI positive (vs. no) (99/1755)	49(49.5)	18(18.2)	15(15.2)	17(17.2)	<0.0001
Experienced violence in the last 12 months (vs. no) (1101/1817)	574(52.1)	290(26.3)	130(11.8)	107 (9.7)	<0.0001
HIV positivity (259/1817)	154(59.5)	49(18.9)	31(12.0)	25 (9.7)	0.004

N = 1817.

Table 3 shows results from the ordered regression model. The odds of experiencing medium to high number of IPV acts after disclosure of HIV test result were higher in women who endorsed more sexual abuse attitudes, experienced more controlling behaviours from their partners, experienced IPV in the 12 months before pregnancy, had been injured by a partner before, were abused in childhood, or reported partners with histories of violence with other people. Women who ever had transactional sex, reported partners who ever tested positive for sexually transmitted infections (STI) or were stopped or prevented from accessing health care by their partners had higher odds of experiencing many acts of IPV after disclosure. However, women who reported that they were currently living with relatives or other members of their family or partner's family in the couple's household had lower odds of reporting high number of IPV events post disclosure. A partially adjusted model that controlled for demographic variables (woman's age, education, marital status), research characteristics (time of HIV test and time of interviews) and violence in the last 12 months shows that IPV after disclosure was associated with HIV serostatus (AOR: 1.88, 1.32–2.68). However, this relationship disappears after adding the behavioural and sexual risk factors in the full model (AOR: 1.09, 0.78–1.52).

**Table 3:** Generalised ordered multiple regression analysis showing factors associated with medium to higher with none to lower IPV (physical, sexual and/or emotional) after disclosing HIV status*.

Variables	Adjusted Odds Ratio	95% CI	p-value
Couple lives with woman's family members (vs. no)	0.68	0.52–0.89	0.006
Couple lives with partner's family members (vs. no)	0.56	0.43–0.73	<0.0001
Experiencing 3–6 controlling behaviours (vs. 0–2)	1.91	1.33–2.73	<0.0001
Woman endorsing 3+ sexual abuse attitudes (vs.0–2)	1.58	1.22–2.03	<0.0001
Partner ever fought with another man (vs. no fighting)	2.31	1.57–3.40	<0.0001
Woman ever injured by a partner (vs.no)	2.39	1.44–3.97	0.001
Child physical and/or sexual abuse (vs. none)	1.66	1.25–2.20	<0.0001
Experienced violence in the last 12 months (vs. no)	6.18	3.84–9.93	<0.00001
Woman stopped/discouraged from accessing antenatal care (vs. encouraged)	1.92	1.28–3.23	0.002
Woman ever had transactional sex (vs. no)	1.82	1.30–2.55	0.001
Partner ever tested to positive to STI (vs. no)	2.03	1.28–3.23	0.003

N = 1817.

*The generalised ordered regression model controlled for woman's age, education, marital status, past experience of violence, time of HIV test, time of interview whether woman tested for HIV before, and number of pregnancies.

CI = confidence interval.

## Discussion

To the best of our knowledge, this is the first study to systematically measure IPV after HIV disclosure to an intimate partner. Our study shows that women overwhelmingly disclosed HIV results to partners - the rate of 93% among pregnant women is amongst the highest reported globally. This study found a high prevalence of physical, sexual or emotional IPV before the pregnancy, after disclosure, as well as high rates of negative reactions from partners immediately after disclosure. It is important to reiterate that IPV after disclosure was high though lower than before pregnancy. It was not surprising that HIV positive women were less likely to disclose their results, but an unexpected finding was the high levels of violence reported irrespective of the HIV result possibly revealing that HIV disclosure adds violence risk to women.

Although only a small proportion of women did not disclose, the findings related to the differences in disclosure between HIV positive and negative women (Unadjusted Odds Ratio [UOR] 0.30, 0.19–0.48) is in agreement with previous studies [8], [9], [23] where women were less likely to disclose their results if they tested positive. This is similar to the outcome of two reviews where the fear of negative effects was identified as an important barrier to disclosure [8], [11]. Similar reports were provided by women during the formative research for this study [18]. Our sample had very high rates of past IPV which could also explain why HIV positive women feared disclosing their results. Another possible explanation for the high levels of disclosure could be related to coercion. About two thirds of the women in a study in South Africa [24] and India [25] indicated that they were coerced to disclose or someone such as a nurse disclosed on their behalf. In our study women reported self-disclosure and we do not know if disclosure was facilitated or coerced by health workers.

We found very high rates of IPV after disclosure of a negative HIV test result. While some studies show that in serodiscordant couples where the male test was negative or unknown, IPV was more common [26], others did not find significant outcome differences between women who tested HIV positive and those who tested HIV negative [3], [9]. Little is known about violence after disclosure of a negative test as most studies focus on HIV positive results although it can be suggested that testing for HIV regardless of the result being positive or negative, may put women at risk of anger and violence from their partners [27].

Many IPV studies have demonstrated the links between gender inequality and experiences of IPV [5]. Our results confirm that unequal gender power relations are a strong predictor of IPV after HIV disclosure. This is illustrated by the positive associations between IPV and higher levels of negative sexual abuse attitudes and male controlling behaviours, including how post natal women access health care. This may all help to explain why women testing negative were also abused. Control of women's reproductive and sexual health decision making was found to be associated with IPV experiences during pregnancy in this study [16] and elsewhere [28]. We conclude that the high levels of IPV reported in the study suggest that violence after disclosure is an extension of previous violence experienced by women, triggered in this instance by having tested for HIV – with the attendant implicit questions about trust and sexual fidelity. The high rates may simply suggest a high level of IPV in more patriarchal relationships where men adhere to more hegemonic masculine roles such as controlling practices and believing they have a right to women's bodies. In such relationships women are also more vulnerable to HIV infection, which could explain higher IPV during pregnancy among HIV+ women. However, our findings also show that a significant proportion of women who had never been abused before pregnancy first experienced abuse after disclosing their status and a significant proportion of them testing HIV positive. Although the sample was small, it helps to show the important contribution that disclosure has on women's experience of violence.

With their focus primarily on HIV positive women, many studies reveal negative outcomes post-disclosure such as disputes, stigma, discrimination, separation, abandonment or being chased away [6], [10]. However if violence was mentioned, systematic measurement was seldom used to define it and sexual and emotional violence are generally ignored [3]. We sought to document both positive and negative reactions including contextually relevant definitions and careful measures of emotional, sexual and physical violence including threat to end the relationship, actually ending the relationship, threat to go out or actually going out with other women, asking about past sexual activities, talking about physical violence. These explicit measures found far higher rates (23.9%) of negative reactions than reported elsewhere [11].

The presence of other people in households may inhibit partner abuse of the woman. Our finding supports previous studies where the presence of other people to support the woman was associated with a decline in IPV [29], [30]. The presence of relatives may also mean that they can physically or emotionally intervene to prevent abuse by the partner. However, further research is needed on the effect of couples living with relatives on violence since extended families can themselves enable violent behaviours in certain contexts [31].

Although HIV positivity was not associated with IPV after disclosure, HIV status was strongly associated with partner's negative reaction immediately after disclosure. Similar findings of no association between HIV status and violence have been reported elsewhere [16], [32]. Risky sexual behaviours of both the woman (transactional sex) and her partner (STI positive test) were associated with IPV after disclosure. This link between risky sexual behaviours and IPV after disclosure adds to the literature on the overlaps of HIV and IPV risk factors [12]. The links between violence, HIV, and “negativity” are complex - there was a strong association between HIV status and reported violence in the unadjusted analysis and partially adjusted model which disappeared after controlling for behavioural and sexual variables- but the association between HIV serostatus and negative immediate reaction remains very strong. This shows the complexity of high risk of informing the partner. The strong association between HIV status and negative reaction helps us to further understand the difficulties that women face when disclosing HIV positive status. HIV testing and conselling programmes must find ways to minimise abuse immediately after disclosure and in the medium to long term. Special focus during counselling must be on enskilling women on how best to disclose to partners to minimise negative reactions and violence. Active involvement of the men in antenatal and postnatal care with their partners may help reduce difficulties associated with disclosure since both partners will receive their test results from the health worker together.

The study has limitations. Firstly, the study was cross sectional and we cannot draw causation inferences based on its cross sectional nature. Although we asked women about abuse after disclosing HIV status, we could have been more specific on whether participants perceived the violence as directly related to the disclosure or whether violence is a normative part of their lives with their partner. Violence could also have been a result of merely testing for HIV without partner's consent since 31.5% of participants who tested negative also reported abuse. There could be other triggers of the violence but it is likely that most motivations are rooted in male domination given normative gender roles and men's belief that they have a right to discipline women. Disclosure may have been one of many possible triggers given the high levels of reported violence before the pregnancy. The HIV status of the male partners was also not known, so we were unable to compare IPV by serostatus concordancy or discordancy. Another limitation is of possible confounding in the measurement of IPV after disclosure because violence after disclosure may be closely linked to the generally high levels of violence reported in the study [16]. However, the assessment of partner's reaction to disclosure also showed higher levels of negative reaction which is strongly associated with HIV status, suggesting a strong link between HIV, disclosure and violence. This could also suggest a strong link between HIV, gender inequality and violence, making negative reactions to disclosure the outcome of underlying higher gender inequalities in relationships where women are found HIV+. Assessing IPV in post natal care excludes women who did not attend postnatal care but were otherwise abused during pregnancy. This means the results may not be generalised to women other than those attending postnatal care.

## Conclusions

The study shows that IPV after disclosure was lower than before the pregnancy. Our study also shows that violence is ubiquitous in these women's lives irrespective of their HIV status. This may have contributed to the disappearance of the association between HIV and IPV after disclosure in the adjusted model. Longitudinal and qualitative studies are needed to further understand this complex relationship. This finding raises a concern about two conflicting public health priorities: the promotion of HIV testing is clearly critical for controlling HIV and especially for linkage to care, but our study has shown that it is also associated with vulnerability to exposure to IPV and negative reactions. This more than ever points to the need to combine HIV and IPV-informed interventions such as safe disclosure and attentive approaches to male involvement within the health sector. Adapting successful health sector prevention interventions on gender based violence relevant to local situations could help to reduce violence related to HIV testing and disclosure [33].

The study demonstrates the interconnectedness of IPV, HIV risk behaviours and women's HIV status. Research is needed to assist in developing interventions in resource-poor settings which may assist women in disclosing their status without further creating harm. We found that negative outcomes including violence occur both immediately (within three days) and some time after disclosure. Promoting HIV disclosure will remain a core component of the fight against HIV. More attention must be given to the gendered nature - and consequences - of disclosure. More sensitivity on the endemic nature of IPV and focus on engaging both women and men in preventing such violence in general and negative and violent reactions after disclosure in particular is needed.
